# Enhancing Resistance to *Enterococcus faecalis*: Immunobiotic *Lactiplantibacillus plantarum* Strains as a Strategy for Malnourished Hosts

**DOI:** 10.3390/nu17111770

**Published:** 2025-05-23

**Authors:** María Daniela Vera, Lorena Paola Arce, Melisa Florencia Müller, Fernanda Raya Tonetti, Ramiro Ortiz Moyano, Héctor Luis Blanco, Haruki Kitazawa, María Guadalupe Vizoso-Pinto, Julio Villena

**Affiliations:** 1 Infection Biology Laboratory, Instituto Superior de Investigaciones Biológicas (INSIBIO), CONICET-UNT, Tucumán 4000, Argentina; danielavera951@fm.unt.edu.ar (M.D.V.); lore.arce.oaq@fm.unt.edu.ar (L.P.A.); melimuller.90@fm.unt.edu.ar (M.F.M.); hector.blanco@conicet.gov.ar (H.L.B.); 2 Laboratorio Central de Ciencias Básicas, Facultad de Medicina, Universidad Nacional de Tucumán, Tucumán 4000, Argentina; 3 Laboratory of Immunobiotechnology, Reference Centre for Lactobacilli (CERELA-CONICET), Chacabuco 145, Tucumán 4000, Argentina; frayatonetti@gmail.com (F.R.T.); rortiz@cerela.org.ar (R.O.M.); 4 Food and Feed Immunology Group, Laboratory of Animal Food Function, Graduate School of Agricultural Science, Tohoku University, Sendai 980-8572, Japan; 5 Livestock Immunology Unit, International Education and Research Center for Food and Agricultural Immunology (CFAI), Graduate School of Agricultural Science, Tohoku University, Sendai 980-8572, Japan

**Keywords:** *Enterococcus faecalis*, *Lactiplantibacillus plantarum*, malnourished, immunobiotic, probiotic

## Abstract

**Background:** *Enterococcus faecalis* causes serious opportunistic infections in patients with weakened immune defenses, such as individuals suffering from malnutrition. We investigated the effects of *Lactiplantibacillus plantarum* MPL16 and CRL1506 on the resistance to *E. faecalis* infection in mice immunosuppressed by protein malnutrition. **Methods:** Male BALB/c mice received a protein-deficient diet for 21 days, followed by a 7-day renourishment period with a balanced conventional diet (BCD) with or without lactobacilli supplementation. Malnourished controls (MN) and renourished mice were treated with gentamicin for 3 days and then challenged orally with *E. faecalis* 102. Infection levels in the gut, liver, spleen, and blood, intestinal tissue damage, and the cytokine response were evaluated 2 days after the challenge. **Results:** Malnourished mice had an impaired ability to produce pro-inflammatory cytokines (TNF, IFN-γ, IL-1β, IL-6, IL-17, and KC) and the regulatory IL-10 in response to the infection compared to mice in the BCD group. The imbalance of inflammatory and regulatory mediators in MN mice favors colonization and invasion by *E. faecalis* and increases tissue damage, making the disease more severe than in animals renourished with BCD. Supplementing the BCD with *L. plantarum* strains significantly enhanced resistance to *E. faecalis* 102 infections, as evidenced by a marked reduction in bacterial loads and intestinal damage. The effect of lactobacilli was associated with enhanced levels of IFN-γ, IL-6, and IL-10 and reduced TNF, IL-1β, IL-17, and KC. **Conclusions:** Given their efficacy in enhancing host resistance, these *Lactiplantibacillus* strains hold great promise as a preventive strategy against *E. faecalis* infections in susceptible individuals.

## 1. Introduction

*Enterococcus faecalis* is a commensal microorganism that inhabits the gastrointestinal tract of mammals without inducing adverse effects. However, this bacterium can cause opportunistic infections in immunocompromised hosts or patients undergoing prolonged antibiotic use [1]. In these cases, this bacterium can play a dual role as both a commensal and a pathogen. *E. faecalis* efficiently colonizes the intestinal mucosa; under certain conditions, it overgrows and can invade the host’s tissues. This bacterium can adapt its metabolic gene expression to the microenvironmental conditions of the intestinal tract [2] and pass from the intestinal lumen to the lamina propria through its internalization in intestinal epithelial cells (IECs) [3,4]. Although enterococcal virulence factors are necessary for the colonization of the intestinal epithelium, the status of the host is also crucial for the onset of infectious disease since alterations of the intestinal barrier, microbiota, and/or the immune system can enable the overgrowth and invasion of enterococci. Research in mice has shown that antibiotics capable of inducing dysbiosis of the intestinal microbiota, such as cephalosporins [5], clindamycin [6], and vancomycin [7], can promote *E. faecalis* overgrowth in the gut and its translocation into the bloodstream. The intestinal microbiota can control the overgrowth of *E. faecalis* through its metabolic activity, such as the production of antimicrobial substances or the modulation of metabolites in the intestinal lumen [8,9]. On the other hand, early studies demonstrated that the administration of the immunosuppressive drug cyclophosphamide significantly augmented the susceptibility of mice to bacteremia and mortality after the oral challenge with *E. faecalis* [10].

Secondary immunodeficiencies can develop because of several factors, including the use of immunosuppressive compounds, cancer, chronic infections, and inadequate nutrition [11]. Nutrient deficiencies alter the immune defenses, leading to enhanced frequency and severity of infections, while infectious diseases potentiate malnutrition as they often promote malabsorption and diminished food intake [12]. The increased susceptibility of malnourished hosts to pathogens, particularly those causing intestinal and invasive infections, has been attributed to alterations of the intestinal microbiota and barrier and the impairment of the immune system [12]. The high rates of cell division and proliferation within the intestinal mucosa make this tissue particularly vulnerable to the detrimental effects of malnutrition. Protein malnutrition influences caspase-dependent apoptosis of IECs, affecting cell turnover [13]. In humans, villous atrophy, increased intestinal permeability, and crypt hyperplasia were described in protein-malnourished hosts [12]. These histological and functional alterations of the intestinal mucosa by protein malnutrition were also found in pigs, rats, mice, and mouse organoids [14,15,16,17]. Protein malnutrition affects the integrity of the gastrointestinal mucosa and, with reduced gastric acid secretion and altered bile production, leads to enhanced susceptibility to infections. On the other hand, studies in animal models and clinical trials in malnourished children demonstrated that malnutrition affects several arms of the immune system including neutrophil chemotaxis and bactericidal capacity, macrophages phagocytic activity, dendritic cells maturation and the levels of interleukin (IL)-1β, IL-6, tumor necrosis factor (TNF), and complement proteins in blood [12]. Malnutrition also affects immune responses mediated by lymphocytes, inducing alterations in the numbers of T cells in the spleen and lymph nodes and circulating B cells and reducing the levels of secretory immunoglobulin A (IgA) [18]. Impairment in the number and function of B and T cells in Peyer’s patches, along with a reduced ability to produce appropriate IgA and IgM antibodies in response to antigens, has been described in malnourished hosts [19]. All these alterations induced by protein malnutrition on the intestinal microbiota, barrier, and immune system have been described to promote *E. faecalis* infection. However, enterococcal infections in protein-malnourished hosts have not been studied in depth despite of the fact that malnourished children demonstrate the intestinal overgrowth of pathobionts [20], including enterococci strains able to induce enteropathy [21], and that antibiotic treatments are usually included as part of the management of malnourished children, even in the absence of infection [22].

The use of functional foods containing probiotic microorganisms with immunomodulatory capacities (immunobiotics) has been proposed to enhance resistance to infections [23]. Studies in immunocompetent mice demonstrated that immunobiotic strains like *Lactiplantibacillus plantarum* CRL1506 [24,25] and *L. plantarum* MPL16 [26] increase immune defense mechanisms and protect against pathogens. Furthermore, we have previously demonstrated that protein-malnourished mice have impaired defenses against *Salmonella* [27], *Candida albicans* [28], and *Streptococcus pneumoniae* [29] and that the repletion of these immunocompromised hosts with a balanced conventional diet (BCD) supplemented with immunobiotic lactobacilli can accelerate the recovery of the immune system and improve the resistance against them.

Enterococcal infections in malnourished hosts remain poorly studied despite evidence that children with malnutrition often harbor an overabundance of intestinal pathobionts that trigger enteric dysfunction [20], that enterococci isolated from malnourished children can induce enteropathy in malnourished gnotobiotic mice [21], and that a broad-spectrum of antibiotics is routinely recommended in guidelines as part of the management of malnourished children, regardless of the presence of clinical symptoms of infection [22]. We hypothesize that protein malnutrition and antibiotic treatment enhance susceptibility and severity of *E. faecalis* intestinal–invasive infection and that the recovery of malnourished hosts with a dietary intervention including immunomodulatory lactobacilli improves mucosal and systemic defenses against opportunistic enterococci. In this work, we tested these hypotheses by evaluating the resistance of protein-malnourished gentamicin-treated mice to an oral challenge with pathogenic *E. faecalis* 102 and the influence of a BCD supplemented with the immunobiotic strains *L. plantarum* MPL16 or CRL1506 on the resistance to the infection and the immune response in the malnourished hosts.

## 2. Materials and Methods

### 2.1. Microorganisms

*L. plantarum* CRL1506 was obtained from the CERELA culture collection (CERELA-CONICET, Tucumán, Argentina) and had been previously isolated from goat milk [30]. *L. plantarum* MPL16 was obtained from the Food and Feed Immunology Group Culture Collection (Tohoku University, Sendai, Japan) and had been previously isolated from the feces of pigs fed with wakame (*Undaria pinnatifida*), a traditional Japanese fermented food [31]. Lactobacilli (stored at −70 °C) were activated and cultured for 12 h at 37 °C (final log phase) in Man-Rogosa-Sharpe (MRS, Britania, Argentina) broth. The bacteria were harvested by centrifugation at 3000× *g* for 10 min and washed three times with sterile 0.01 mol/L PBS, pH 7.2, and resuspended in sterile 15% non-fat milk.

*E. faecalis* 102 was obtained from the Culture Collection of the Faculty of Biochemistry, Chemistry and Pharmacy of the National University of Tucumán. This strain had been previously isolated from a wound of a 41-year-old patient from the Angel C. Padilla Hospital (San Miguel de Tucumán, Argentina) and was selected in preliminary experiments in malnourished mice because of its ability to induce intestinal–invasive infection. *E. faecalis* 102 was cultured in Brain Heart Infusion (BHI, Britania, Argentina) at 37 °C for 16 h and stored in 50% sterile glycerol at −70 °C for further use.

### 2.2. Survival of Lactic Acid Bacteria to Gastrointestinal Conditions In Vitro

Gastrointestinal (GI) conditions were simulated by following the methods reported before [32,33,34,35]. Briefly, to test the survival to gastrointestinal conditions, lactic acid bacteria (LAB) were grown for 16 h in MRS, washed twice with phosphate-buffered saline (PBS), and suspended in MRS or 15% reconstituted skim milk after centrifugation. Then, LAB were sequentially subjected to 1:1 dilution in a buffer mimicking saliva (NaCl 6.2 g/L; KCl 2.2 g/L; CaCl_2_ 0.22 g/L; NaHCO_3_ 1.2 g/L pH 7.2), a 3:5 dilution in artificial gastric fluid (NaCl 6.2 g/L; KCl 2.2 g/L; CaCl_2_ 0.22 g/L; NaHCO_3_ 1.2 g/L pH 2.5), and a 1:4 dilution in synthetic intestinal fluid (NaHCO_3_ 6.4 g/L; KCl 0.239 g/L; NaCl 1.28 g/L; sodium deoxycholate monohydrate (Sigma-Aldrich, Buenos Aires, Argentina) 0.5%; pancreatin protease (Pankreoflat A.D., Munro, Argentina) (>1900 USP). Samples were incubated under constant shaking for 5, 60, and 120 min to simulate the digestion processes occurring in saliva, gastric fluids, and intestinal fluids, respectively [34,35]. Then, viable LAB cells were serially diluted (10-fold dilutions), plated onto MRS agar plates, and incubated at 37 °C for 48 h under anaerobic conditions. The assay was performed in triplicate twice.

### 2.3. Animals Feeding Procedures and Experimental Infection in Malnourished Mice

**Figure 1:**
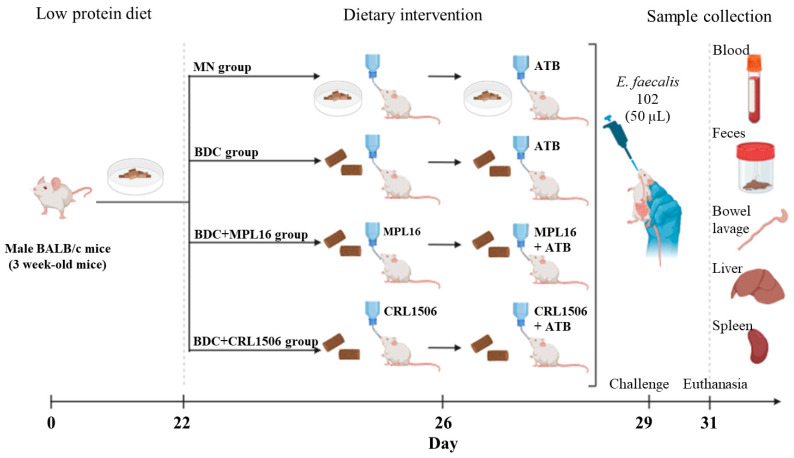
Experimental design. For 21 days, mice were fed a low-protein diet. Two control groups were included: malnourished mice without renourishment (MN group) and mice renourished with a conventional balanced diet (BDC) for 7 days without LAB supplementation (BDC group). The LAB was administered at a dose of 10^8^ cells/mouse/day in drinking water (BDC + MPL16 and BDC + CRL1506 groups). Three days before the challenge with *E. faecalis* (day 26), the mice were treated with gentamicin (1.6 mg/kg) (ATB) supplemented in drinking water. On day 29, BALB/c mice were orally challenged with *E. faecalis* (50 µL, 10^8^ UFC/mL). After euthanasia (day 29), samples were collected (blood, feces, intestinal lavage, liver, and spleen).

Mice and Diet. Three-week-old male BALB/c mice were obtained from CERELA (San Miguel de Tucumán, Argentina). After weaning, the mice were fed a low-protein diet for 21 days. This diet consisted mainly of carbohydrates supplemented with vitamins, essential fatty acids, and minerals [29,36,37]. As shown in Figure 1, mice were randomly divided into four groups of 5 animals each. Two control groups were included: malnourished mice without renourishment (MN group) and mice renourished with a conventional balanced diet (BDC) for 7 days without LAB supplementation (BDC group). The composition of BCD is provided in the Supplementary Table S1. *L. plantarum* MPL16 or *L. plantarum* CRL1506 were administered at a dose of 10^8^ cells/mouse/day in drinking water (BDC + MPL16 and BDC + CRL1506 groups, respectively). Three days before the challenge with *E. faecalis* (day 26), the mice were treated with the antibiotic gentamicin (1.6 mg/kg) (Invitrogen, Dreieich, Germany) [38] supplemented in drinking water (Figure 1). The experimental unit was each animal. The total number of animals used was 20 mice, and none were excluded. The number of animals was decided based on previously published works [28,29,30]. To minimize potential confounders, treatments and challenges were performed in different orders. Samples were blinded for group members analyzing the samples and data. Animals were monitored daily in order to avoid suffering. None of the animals died during the experiment.

Oral challenge with *E. faecalis*. *E. faecalis* 102 was cultured on BHI agar for 18 h; freshly grown colonies were suspended in 5 mL of BHI broth and incubated at 37 °C for 16 h under shaking. The bacterial cells were harvested by centrifugation at 10,000 rpm for 10 min and washed three times with sterile PBS. The cell density was adjusted to 8 × 10^8^ cells/L.

On day 29, mice were orally challenged with a single dose of E. faecalis 102 (50 µL, 10^8^ UFC/mL) and euthanized 48 h post-challenge. The mouse samples (blood, feces, intestinal fluid, liver, and spleen) were collected for subsequent analysis (Figure 1). To minimize animal suffering, predefined humane endpoints were established and approved by the Institutional Animal Care and Use Committee. Mice were monitored at least twice daily for clinical signs of distress, especially during infection. Animals were euthanized if they met any of the following criteria: (1) persistent lethargy or inability to access food or water; (2) severe diarrhea or signs of dehydration, (3) hunched posture, ruffled fur, and inactivity lasting more than 24 h; (4) signs of systemic infection or sepsis, including labored breathing; or (5) self-isolation or failure to engage in normal behaviors.

### 2.4. Counts of E. faecalis Colonies in Mouse Samples

Two days after the challenge with *E. faecalis* (31 days), mouse samples were aseptically collected and suspended in 5 mL of sterile PBS to determine viable *E. faecalis* counts. Fecal samples were disaggregated with a sterile pestle and homogenized in PBS. Bowel lavage was collected by flushing the small intestine with PBS. The liver and spleen were homogenized in 5 mL of sterile peptone water. Blood samples were obtained via cardiac puncture and collected in heparinized tubes.

Mouse samples were serially diluted, plated in duplicate on BHI agar, and incubated for 18 h at 37 °C. Due to the differences in growth kinetics, it was possible to differentiate enterococci from lactobacilli: the colonies of *E. faecalis* were visible and counted (UFC/mL) after the first 24 h, whereas the colonies of *L. plantarum* appeared later, after 48 h.

### 2.5. Determination of Cytokine Concentrations in Serum and Bowel Lavage

To evaluate the local and systemic immune response, as well as the level of inflammation caused by *E. faecalis* 102, cytokine concentration was measured in serum and intestinal lavage samples obtained from malnourished mice treated with LAB and subsequently challenged with *E. faecalis* 102. The concentrations of TNF, interferon (IFN)-γ, IL-1β, IL-6, keratinocyte chemoattractant (KC), IL-10, and IL-17 in serum and bowel lavage were measured using commercially available enzyme-linked immunosorbent assay (ELISA) kits, following the manufacturer’s recommendations (R&D Systems, Minnneapolis, MN, USA).

### 2.6. Histopathology

After 48 h of the challenge with *E. faecalis* 102, euthanasia was performed, and small intestine samples were collected for histological analysis. These samples were stored in 10% formalin (Biopack, Buenos Aires, Argentina) and kept at 4 °C until processing. Samples were first dehydrated by passing them through a series of alcohol solutions (Frau ethyl alcohol 96°, Argentina), with increasing concentrations: two passages through 70% alcohol, one through 80% alcohol, and two through 96% alcohol. The samples were transferred to butyl acetate (Cirarelli, San Lorenzo, Argentina) and a histological clearing agent (Pathoclear^®^Plus, Biopack, Argentina), each step involving 1 h incubation. After incubation, the samples were embedded in paraffin (Biopack, Argentina) through two additional 1 h incubation periods. The inclusion process was followed, during which tissue blocks were assembled. Serial sections were made using a rotary microtome, with section sizes ranging from 3 to 5 microns. Once sectioning was complete, the staining process began. The cut sections, already mounted on slides, were rehydrated and immersed in hematoxylin stain (Biopack, Argentina) for 5 min. They were rinsed with running water and subsequently immersed in an eosin stain (Biopack, Argentina) for another 5 min. After staining, the samples were again passed through alcohol solutions, butyl acetate, and a clearing agent. Finally, the mounting process was carried out by placing a drop of synthetic Canadian balsam (Biopack, Argentina) on the slide and covering it with a cover slip. Once prepared, the samples were examined under an optic microscope (Leica, Wetzlar, Germany).

### 2.7. Statistical Analysis

Experiments were performed in duplicates, and results were expressed as mean  ±  standard deviation (SD). Statistical analyses were performed using Prism 8.0 (GraphPad software, San Diego, CA, USA). After verification of the normal distribution of data, one-way ANOVA was used. Tukey’s test (for pairwise comparisons of the means) was used to test for differences between the groups. Differences were considered significant at *p*  <  0.05. Graphics were created using BioRender (https://www.biorender.com/, May 2025).

### 2.8. Ethical Statement

Experiments with mice were performed in accordance with the guidelines for the care and use of laboratory animals approved by the CERELA-CONICET Animal Care and Ethics Committee, protocol number CRL-CICUAL-IBT-2024/2A, approved 24 June 2024.

## 3. Results

### 3.1. Survival to In Vitro Gastrointestinal Conditions

The survival of *L. plantarum* strains MPL16 and CRL1506 under simulated GI conditions was evaluated. ON cultures were suspended in either fresh MRS broth or 15% reconstituted skimmed milk and subsequently subjected to sequential exposure to simulated saliva, gastric juice, and intestinal juice.

**Figure 2:**
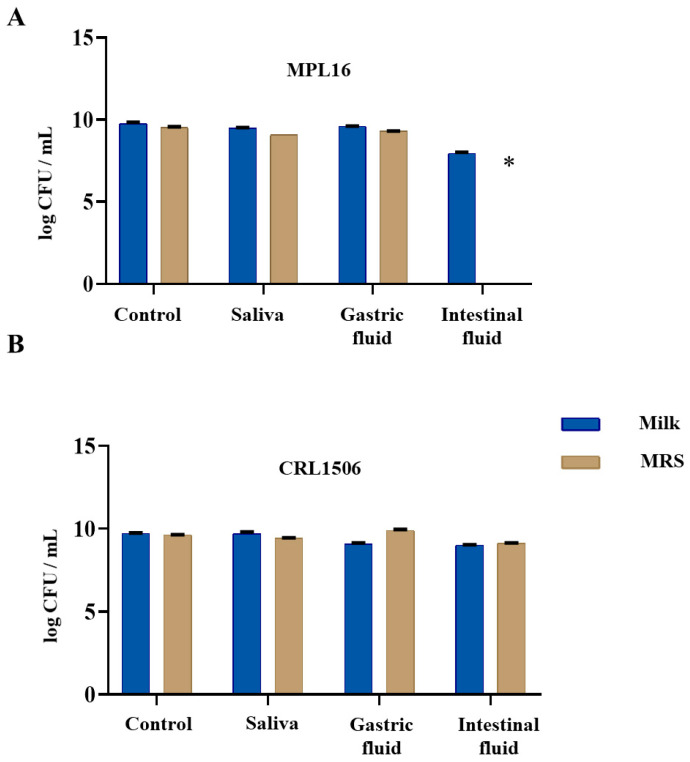
Survival of LAB in vitro gastrointestinal conditions. Effect of the resistance of *L. plantarum* (**A**) MLP16 and (**B**) CRL156 strains, previously suspended in MRS or 15% reconstituted skim milk, to an artificial gastrointestinal passage (simulated saliva, gastric juice, and intestinal juice solutions). The viable LAB cells were serially diluted (10-fold dilutions), plated onto MRS agar plates, and incubated at 37 °C for 48 h under anaerobic conditions. Results are presented as mean ± SD. Statistical differences between the groups were indicated as * *p* < 0.05.

When MPL16 was cultured in MRS, it survived the passage over simulated saliva and gastric juice, maintaining the initial bacterial counts (~10^9^ CFU/mL) (Figure 2A). However, exposure to simulated intestinal juice resulted in the complete loss of viability. To enhance its resistance, MPL16 was suspended in 15% reconstituted milk and retested. Under these conditions, MPL16 maintained the CFU levels at ~10^9^ CFU/mL after exposure to saliva and gastric juice. However, in intestinal juice, CFU levels decreased by two log units, indicating sensibility but improved protection (Figure 2A).

In contrast, CRL1506 exhibited robust resistance to simulated GI conditions regardless of whether it was tested in MRS or 15% reconstituted milk, maintaining CFU levels at 10^9^ CFU/mL throughout the experiment (Figure 2B).

Given the ability of both strains to resist gastrointestinal passage when suspended in 15% reconstituted milk, this medium was selected for subsequent in vivo assays. Specifically, the conditions were applied in a murine model of immunosuppression induced by malnutrition (Figure 1).

### 3.2. Effect of LAB on Resistance to Infection by E. faecalis

**Figure 3:**
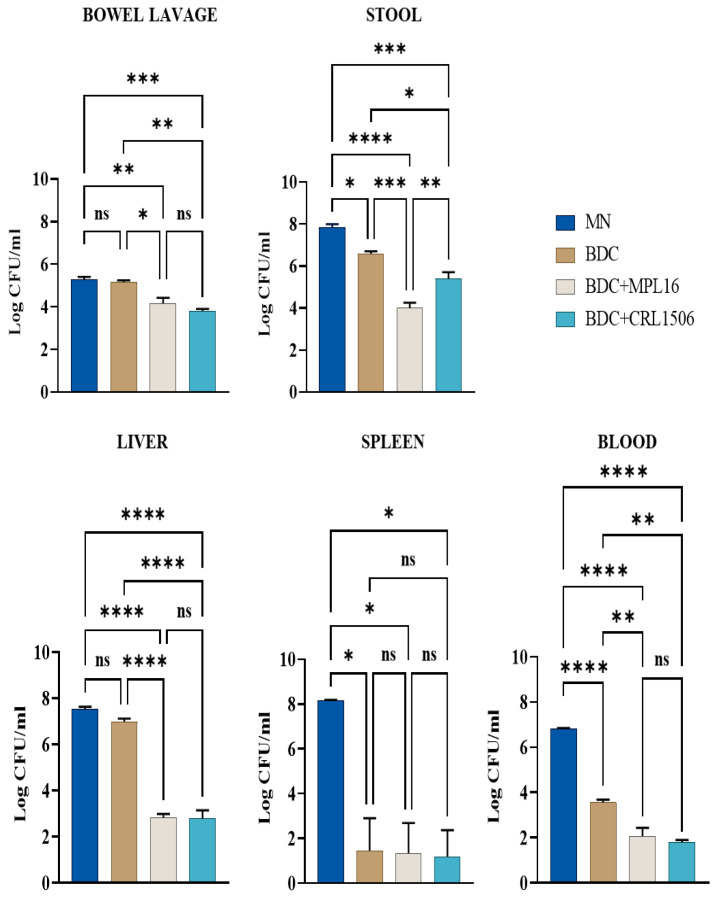
Effect of LAB on resistance of malnourished mice to *E. faecalis* infection. After malnutrition, mice were renourished with a conventional balanced diet (BDC) alone (BDC group), or supplemented with LAB (BDC + MPL16 and BDC + CRL1506 groups), and malnourished mice without renourishment (MN group). The mice were subsequently challenged with a single dose of *E. faecalis* (50 µL, 10^8^ UFC/mL) and euthanized 48 h post-challenge. Post-euthanasia, *E. faecalis* was counted in mouse samples (stool, blood, spleen, liver, and bowel lavage). Results are presented as mean ± SD. Statistical differences between the groups were indicated as follows: * *p* < 0.05, ** *p* < 0.01, *** *p* < 0.001, **** *p* < 0.000, and not significant (ns) > 0.05.

To evaluate the impact of renourishment with LAB (MPL16 or CRL1506) on resistance to *E. faecalis* infection, viable bacterial counts were assessed in different organs following the oral challenge with the pathogen. None of the animals showed any of the endpoint criteria. The BDC mice exhibited significantly lower *E. faecalis* loads in stool, blood, and spleen compared to the control group (MN group) (Figure 3). However, administering MPL16 or CRL1506 during renourishment (BDC + MPL16 and BDC + CRL1506 groups) significantly reduced the bacterial burden in all organs tested. Further, statistical analysis revealed that both MPL16 and CRL1506 treatments led to a marked decrease in *E. faecalis* counts in the liver (*p* < 0.0001) and spleen (*p* < 0.01). Additionally, bacterial levels in the blood and feces were significantly lower in the immunobiotic-treated groups than in the BDC group (*p* < 0.01) (Figure 3). These results suggest that MPL16 and CRL1506 supplementation during renourishment improves systemic resistance to *E. faecalis* infection.

### 3.3. Effect of LAB on Serum Cytokine Levels After Infection with E. faecalis 102

Treatment with MPL16 and CRL1506 strains significantly reduced serum levels of the inflammatory cytokines TNF, IL-1β, and IL-6. No significant differences were observed in TNF levels in intestinal fluid samples between LAB-treated and *E. faecalis*-infected mice. However, intestinal IL-6 levels were elevated following LAB treatment.

**Figure 4:**
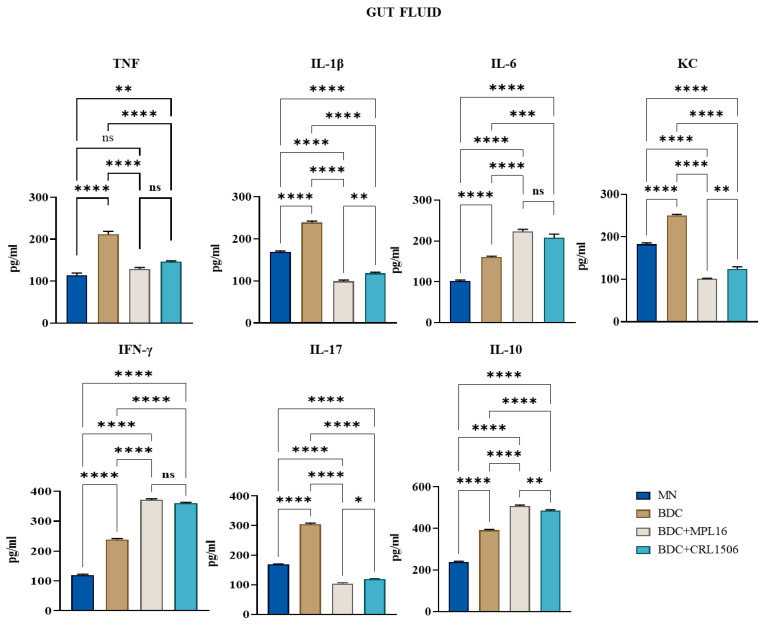
Effect of LAB on intestinal cytokine levels after infection with *E. faecalis* 102. After malnutrition, mice were renourished with a conventional balanced diet (BDC) alone (BDC group) or supplemented with LAB (BDC + MPL16 and BDC + CRL1506 groups), and malnourished mice without renourishment (MN group). The mice were subsequently challenged with a single dose of *E. faecalis* 102 (50 µL, 10^8^ UFC/mL). Two days post-challenge with *E. faecalis* 102, a serum sample was collected to determine IL-6, IL-8, IL-1, TNF, IFN-γ, IL-10, and IL-17 levels. Results are presented as mean ± SD. Statistical differences between the groups were indicated as follows: * *p* < 0.05, ** *p* < 0.01, *** *p* < 0.001, **** *p* < 0.000, and not significant (ns) > 0.05.

**Figure 5:**
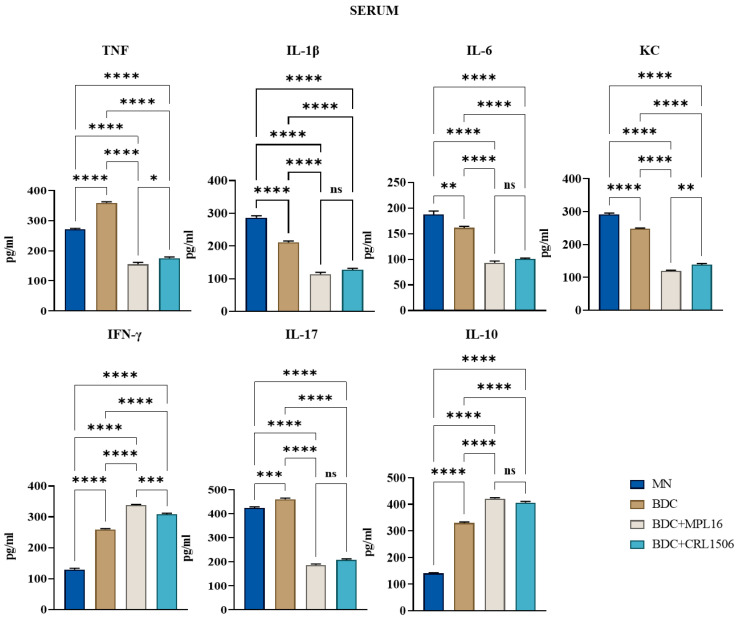
Effect of LAB on serum cytokine levels after infection with *E. faecalis* 102. After malnutrition, mice were renourished with a conventional balanced diet (BDC) alone (BDC group), or supplemented with LAB (BDC + MPL16 and BDC + CRL1506 groups), and malnourished mice without renourishment (MN group). The mice were subsequently challenged with a single dose of *E. faecalis* 102 (50 µL, 108 UFC/mL). Two days after the challenge, IL-6, IL-8, IL-1, TNF, IFN-γ, IL-10, and IL-17 levels were determined in gut fluid. Results are presented as mean ± SD. Asterisks indicate significant differences between the indicated groups (* *p* < 0.05, ** *p* < 0.01, *** *p* < 0.001, **** *p* < 0.000, and not significant (ns) > 0.05).

Additionally, the effects of LAB supplementation on IFN-γ and IL-10 levels were assessed. Treatment resulted in increased concentrations of both immunological factors in intestinal fluid and serum samples compared to infected animals. Furthermore, LAB-treated mice exhibited lower intestinal and serum levels of KC and IL-17 than those challenged with the *E. faecalis* strain. (Figure 4 and Figure 5).

### 3.4. Restoration of Small Intestine Histology During Re-Nutrition

**Figure 6:**
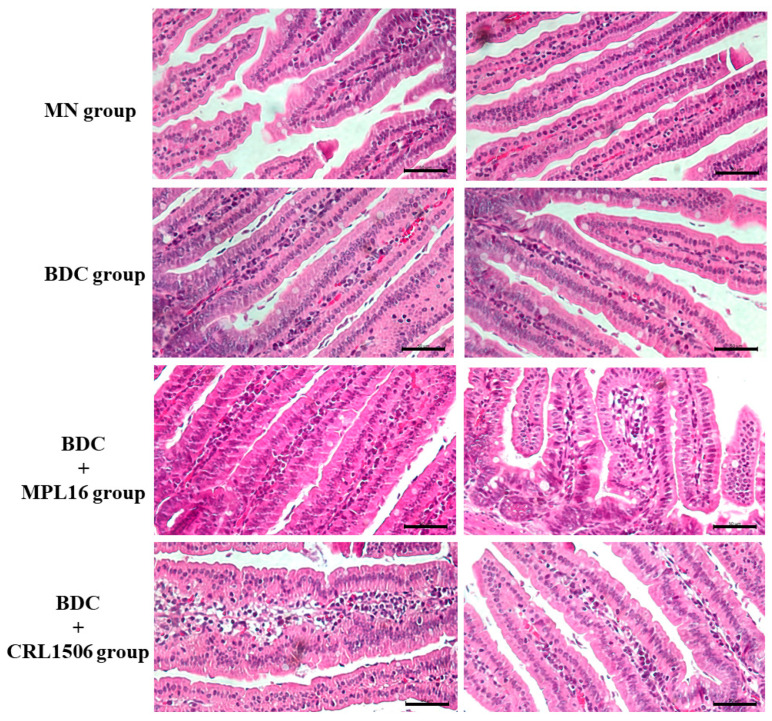
Effects of a low-protein diet and renourishment with LAB on the intestinal mucosal morphology in mice after infection with *E. faecalis* 102. The malnourished mice without renourishment, MN group, were renourished with a conventional balanced diet (BDC) alone, BDC group, or supplemented with LAB, BDC + MPL16, and BDC + CRL1506 groups, and subsequently challenged with *E. faecalis.* Histological analysis of the intestine mucosal showed thinner villi in the MN group compared to the BDC group. Additionally, groups treated with LAB (MPL16 and CRL1506) exhibited increased immune cell recruitment compared with MN and BDC groups. Scale bar = 50 μm.

The histological structure of the small intestine was altered in the MN group, with shorter and thinner intestinal villi compared to the BDC group (Figure 6). Administration of immunobiotic strains during the renourishment period partially restored these histological features (Figure 6). Mice that received MPL16 and CRL1506 also showed a remarkable recruitment of immune cells in the lamina propria.

## 4. Discussion

*E. faecalis* has been associated with life-threatening infections in patients with chronic antibiotic use or a compromised immune status [8,9]. In this regard, it was shown that the treatment of mice with antibiotics together with the immunosuppressive drug cyclophosphamide significantly augmented the susceptibility of animals to *E. faecalis* bacteremia after oral challenge with the pathogen, leading to increased mortality [10]. In this work, the immunosuppression induced by protein deficiency in the diet, together with the oral treatment with gentamicin, allowed *E. faecalis* intestinal–invasive infection of the malnourished mice. Male mice were selected as the experimental model for probiotic supplementation in the context of enterococcal infection due to epidemiological and biological factors that reflect increased male vulnerability to undernutrition and infection. Males are more affected by undernutrition, particularly in severe cases and low-resource settings, and are generally more vulnerable to infectious diseases, likely due to differences in immune and endocrine function [39]. Clinically, vancomycin-resistant enterococci (VRE) bloodstream infections show a male predominance, suggesting sex-specific factors in disease susceptibility. These considerations justify the use of male mice to better reflect the human populations most at risk [40].

We observed that MN mice have significantly higher enterococcal loads in the intestine as well as in the liver, blood, and spleen compared to mice renourished with BCD, indicating the higher susceptibility of malnourished animals to *E. faecalis* intestinal–invasive infection. The results presented here, and the studies performed previously in this murine malnutrition model allow us to speculate that the reduced resistance of malnourished mice to *E. faecalis* infection could be associated with: (i) the dysbiosis of the intestinal microbiota, (ii) the damage of the intestinal epithelial barrier and (iii) the impairment of the mucosal and systemic immune systems.

Intestinal microbiota dysbiosis is a hallmark of malnutrition. Protein malnutrition can affect gastric acid secretion and alter bile production, which contribute to intestinal microbiota dysbiosis [41]. Although in our model of protein-malnourished mice, the changes in the intestinal microbiota were not investigated using culture-independent techniques, it was previously reported that these malnourished mice had significantly lower numbers of lactobacilli and anaerobic microorganisms and higher numbers of Gram-negative bacilli in both the small intestine and colon compared to well-nourished animals [42]. These results are in line with works describing alterations of the intestinal microbiome in mice receiving diets with protein deficiencies [43,44]. In addition, studies conducted in gnotobiotic C57BL/6 mice showed that *E*. *faecalis* can pass from the intestinal lumen to the lamina propria through internalization via IECs [3,4]. Protein malnutrition impairs the IECs’ turnover [13], and induces villous atrophy, increased intestinal permeability, and crypt hyperplasia [12,14,15,17,42]. The structural and ultrastructural alterations of the intestine have been reported previously in the model of protein-malnourished mice used in this work [42,45,46], which probably allowed *E. faecalis* intestinal colonization and invasion. Further studies of the gut microbiota using novel omics tools and the alteration of the intestinal epithelium in our protein malnutrition model in the context of *E. faecalis* infection could provide further insight into the role of these intestinal barriers in the increased susceptibility of malnourished mice to *E. faecalis* infection. These are interesting topics for future research.

We observed that MN mice had lower levels of TNF, IL-1β, IL-6, KC, IL-17, IFN-γ, and IL-10 in the intestine compared to BCD mice after the enterococcal infection. In addition, MN animals had lower concentrations of TNF, IL-17, IFN-γ, and IL-10 as well as higher levels of IL-1β, IL-6, and KC in blood. The reduced capacity of protein-malnourished mice to produce inflammatory factors in response to *E. faecalis* infection is in line with our previous studies evaluating their immune response to other pathogens, such as *C. albicans* [28] and *S. pneumoniae* [29]. Pneumococcal infection can enhance the concentrations of TNF, IL-1β, IL-6, IFN-γ, and IL-10 in the respiratory tract and blood of immunocompetent well-nourished mice, while in protein-malnourished the levels of those immune mediators are significantly lower [47]. Similarly, the intraperitoneal challenge of well-nourished and protein-malnourished mice with *C. albicans* increased the levels of TNF, IL-6, and IFN-γ in the peritoneal cavity as well as TNF, IL-6, IFN-γ, IL-17, and IL-10 concentrations in blood. However, we observed significantly lower values of all these cytokines in MN animals than in normal controls [28]. It was reported that children with severe malnutrition often have a diminished ability to produce acute-phase proteins and pro-inflammatory mediators like TNF, IL-1β, and IL-6 [48,49] while peritoneal macrophages from protein-deficient mice produce low levels of TNF in response to lipopolysaccharide challenge [50]. Of note, macrophages from protein-deficient mice responded to TNF stimulation with lower concentrations of IL-1β and IL-12 than cells obtained from control animals [51]. However, it was also shown that uninfected malnourished children have high levels of circulating TNF [52]. Furthermore, malnourished mice infected with the enteric pathogen *Salmonella* Typhimurium had higher TNF, IL-6, MCP-1, and IFN-γ levels in the livers than control-infected mice [53]. This discordance may be due to differences in malnutrition severity and the distinct inflammatory challenges. Even though increases or decreases in immunological mediators have been recorded for malnourished hosts, their response has been consistently described as being inappropriate for eliminating pathogens. In fact, the different intestinal and serum cytokine profiles of malnourished mice in response to *E. faecalis* infection were associated with higher bacterial loads in the intestine and internal organs and with their dissemination into blood, resembling our previous results with *C. albicans* and *S. pneumoniae* [28,29].

We have previously reported quantitative and qualitative impairments of phagocytes in our model of protein malnutrition. MN mice have lower numbers of blood neutrophils [47] and reduced phagocytic and microbicidal activities in peritoneal and respiratory macrophages [28,29]. The recruitment of neutrophils into the peritoneal cavity, as well as their peroxidase activity after the challenge with *C. albicans,* was reduced in protein-malnourished mice compared to normal controls [28]. They also showed a significant impairment in the recruitment of neutrophils and macrophages into the respiratory tract after the infection with *S. pneumoniae* [29], which was related to qualitative alterations of immune cells in blood and bone marrow [47]. In line with these findings, we observed that malnourished mice had a reduced ability to recruit immune cells to intestinal tissue after the challenge with *E. faecalis* compared to mice repleted with BCD. Then, the failure in the establishment of this immunological phagocytic and microbicidal barrier in the gut of protein-malnourished mice could contribute to the invasive enterococcal infection.

The production of regulatory cytokines during the course of infections is of importance for avoiding dysregulated inflammation and host damage. In this work, although protein malnourished mice had lower levels of pro-inflammatory cytokines and chemokines in the intestine, they also showed higher levels of IL-1β, IL-6, and KC in blood compared to mice in the BCD groups. Of note, the levels of the regulatory cytokine IL-10 were significantly lower in both the intestine and blood in malnourished mice than in animals repleted with BCD after the enterococcal infection. Thus, malnourished mice showed an imbalance of inflammatory mediators in the different compartments along with a notable impairment in their ability to regulate the inflammatory response. This imbalance of immune mediators in protein malnourished mice would allow the colonization and invasion of *E. faecalis* and the inflammatory-mediated tissue damage, making the disease more severe than in animals repleted with BCD.

We demonstrated here that BCD supplemented with *L. plantarum* MPL16 or *L. plantarum* CRL1506 was more efficient than BCD alone to improve the resistance and modulate the immune response to *E. faecalis* infection in repleted malnourished animals. This is in line with our previous studies showing that immunomodulatory lactobacilli can accelerate the recovery of immune defense mechanisms when used as supplements in repletion diets [28,29]. Protein-malnourished mice renourished with BCD and the MPL16 or CRL1506 strains had lower intestinal, liver, spleen, and blood loads of enterococci than mice receiving only BCD. This distinct resistance to *E. faecalis* infection could be associated with the different cytokine profiles found in both the intestine and serum. Previously, we showed that protein-malnourished mice treated with both BCD and BCD supplemented with the CRL431 strain enhanced the concentrations of TNF, IL-6, and IFN-γ in the peritoneal cavity, blood, liver, and spleen in response to *C. albicans* infection [28]. Cytokine levels were higher than the concentrations observed in malnourished controls, but only mice renourished with the immunomodulatory lactobacilli reached the values of well-nourished animals. Interestingly, the levels of IL-10 were higher in mice treated with BCD and *L. casei* CRL431 than in well-nourished mice. In addition, both immunomodulatory CRL431 and CRL1505 strains improved the production of TNF, IL-1β, IL-6, and IFN-γ in the respiratory tract compared to malnourished and BCD-treated mice, after the pneumococcal infection. Interestingly, IL-10 production in response to *S. pneumoniae* was higher in BCD and CRL341-treated malnourished mice than in controls but similar to well-nourished controls in mice receiving the BCD and the CRL1505 strain [47]. The results of this study contrast with those of previous works, since protein-malnourished mice renourished with BCD and the MPL16 or CRL1506 strains had lower TNF, IL-1β, KC, and IL-17 in the gut and blood, while IL-6 was higher in the intestine but lower in blood, compared with animals treated only with BCD. In addition, we observed improved levels of IFN-γ and IL-10 in mice of the BCD + MPL16 and BCD + CRL1506 groups than in the BCD mice. These differences in the effect of lactobacilli on cytokine profiles of malnourished hosts may be due to three combined factors: the pathogen used, the initial anatomical site of infection, and the different strains of immunomodulatory lactobacilli used.

Malnourished mice treated with BCD and the MPL16 or CRL1506 have reduced levels of inflammatory cytokines and enhanced IL-10, a balance that could be involved in the protection of inflammatory-mediated intestinal barrier damage during the enterococcal infection. The production of inflammatory mediators in response to *E. faecalis* is important to eliminate the pathogen. However, the inflammatory response must be tightly regulated to avoid the host’s tissues. As mentioned before, IL-10 deficiency can lead to enhanced inflammatory disease in mice infected with *E. faecalis* [54,55]. On the other hand, TNF can disrupt epithelial barrier function through the activation of NF-κB and MAPK pathways [56] and the modulation of zonulin-1 [57] and cytoskeletal filaments [58].

Our findings reveal that malnourished mice treated with BCD and the immunobiotic strains *L. plantarum* MPL16 or CRL1506 exhibit increased levels of IL-6 and IFN-γ, which help reinforce gut epithelial and immune barriers. IFN-γ production by phagocytes plays a key role in protecting against *E. faecalis* invasion, while IL-6 is essential for intestinal homeostasis, tight junction integrity, and epithelial cell proliferation [59,60,61]. In immunocompetent hosts, both *L. plantarum* strains enhanced IL-6 and IFN-γ levels, improved the recruitment of immune cells to the intestinal mucosa, and modulated IECs and antigen-presenting cell responses. These strains can upregulate IFN-β, IL-6, and TNF in IECs [25,26]. Additionally, *L. plantarum* CRL1506 stimulated antigen-presenting cells in Peyer’s patches, boosting IL-1β, IFN-γ, IL-12, and activation markers (MHC-II and CD80/86), which correlated with an enhanced Th1 response [25,62]. Then, it is tempting to speculate that lactobacilli exerted similar effects on IECs and phagocytes in malnourished mice treated with BCD. While our study highlights the immunomodulatory benefits of *L. plantarum* MPL16 and CRL1506, a limitation is that cytokine levels were assessed at a single time point after *E. faecalis* challenge. Future studies should investigate the kinetics of immune responses and the specific roles of IECs and antigen-presenting cells in immunobiotic-induced protection.

## 5. Conclusions

This work provides evidence that protein deficiency and antibiotic treatment compromise gut defenses, facilitating the intestinal colonization and invasion of *E. faecalis*. This underscores the need for cautious antibiotic use during malnutrition recovery, given the rise in antibiotic-resistant enterococci. Further, short-term BCD repletion alone was insufficient to restore defenses against *E. faecalis* infection, aligning with previous findings that immune recovery requires at least 21 days of BCD treatment [29]. However, supplementing BCD with *L. plantarum* MPL16 and CRL1506 accelerated immune recovery and improved resistance to infection by modulating systemic and gut cytokine profiles. These results suggest that immunobiotic lactobacilli could be integrated into nutritional rehabilitation strategies for malnourished children to enhance immune function and protect against opportunistic infections.

## Data Availability

The data that support the findings of this study are available from the corresponding author upon reasonable request.

## Supplementary Materials

The following supporting information can be downloaded at https://www.mdpi.com/article/10.3390/nu17111770/s1. Table S1: Composition of the conventional balanced diet and low-protein diet. Table S2: LAB survival data under in vitro gastrointestinal conditions. Table S3: Data on the effect of LAB on the resistance of malnourished mice to *E. faecalis* infection. Table S4: Data on the effect of LAB on intestinal cytokine levels after infection with *E. faecalis* 102. Table S5: Data on the effect of LAB on serum cytokine levels after infection with *E. faecalis* 102.
