# Supplementary material for: Enhancing Resistance to Enterococcus faecalis: Immunobiotic Lactiplantibacillus plantarum Strains as a Strategy for Malnourished Hosts
Source: Nutrients. 2025 May 23;17(11):1770. doi: 10.3390/nu17111770 (PMC12157614; doi:10.3390/nu17111770)
Supplement: Supplementary file 1 [file nutrients-17-01770-s001.zip › nutrients-3616054-supplementary.pdf]

Supplementary table 1. Composition of the conventional balanced diet and low-protein diet

Ingredient	Conventional balanced diet g/kg	Low-protein diet g/kg
Water	120	120
Protein	230	<10
Carbohydrate	538	758[1]
Lipids	50	50
Vitamin mix[2]	22[2]	22
Mineral mix[3]	40[3]	40

1-Protein-free corn flour.

2-Vitamin mix (# 905454, ICN Biomedicals Argentina) g/kg of mixture: dl-α-tocopherol, 5.0; p-aminobenzoic acid, 5.0; ascorbic acid, 45.0; biotin, 0.02; retinyl acetate, 4.5; vitamin B-12, 0.00135; calcium pantothenate, 3.0; choline chloride, 75.0; cholecalciferol, 0.25; folic acid, 0.09; inositol, 5.0; menadione, 2.25; niacin, 4.5; pyridoxine hydrochloride, 1.0; riboflavin, 1.0 thiamine hydrochloride, 1.0; and sucrose, finely powdered, 847.38865.

3-Mineral mix (# 902844 ICN Biomedicals Argentina) g/kg of mixture: sodium chloride, 167; potassium phosphate dibasic, 322; calcium carbonate, 300; magnesium sulfate, 102; calcium phosphate monobasic, 75; ferric citrate, 27.5; $MnSO_4$. H_2O, 5.1; potassium iodide, 0.8; CuSO4. $5H_2O$, 0.3; zinc chloride, 0.25; and $CoCl_2.6H_2O$, 0.05.

Supplementary table 2: LAB survival data under *in vitro* gastrointestinal conditions

Treatment conditions	15% reconstituted skim milk Group A		Man Rogosa and Sharpe (MRS) Group B	
	A:1	A:2	B:1	B:2
Control	9,69897	9,82351	9,47712	9,55321
Saliva	9,477121	9,51240	9,07918	9,08127
Gastric fluid	9,60206	9,58432	9,30103	9,28324
Intestinal fluid	8,00000	7,89653	0	0

Supplementary table 3. Data on the effect of LAB on the resistance of malnourished mice to *E. faecalis* infection

**BOWEL LAVAGE**	Group A	Group B	Group C	Group D
	MN	BDC	BDC+MPL16	BDC+CRL1506
	5,53782	5,30103	4,17609	3,69897
	5,16137	5,00000	4,60206	4,00000
	5,07918	5,17609	3,69897	3,69897

**STOOLS**	Group A	Group B	Group C	Group D
	MN	BDC	BDC+MPL16	BDC+CRL1506
	7,64836	6,47712	4,20412	5,00000
	8,15836	6,47712	4,30103	6,00000
	7,67209	6,81291	3,47712	5,17609

**LIVER**	Group A	Group B	Group C	Group D
	MN	BDC	BDC+MPL16	BDC+CRL1506
	7,73639	6,92064	3,11351	3,49099
	7,46239	6,75967	2,76447	2,49485
	7,36173	7,23552	2,61978	2,35654

**SPLEEN**	Group A	Group B	Group C	Group D
	MN	BDC	BDC+MPL16	BDC+CRL1506
	8,21352	4,352183	0	3,55284
	8,12222	0	4,02996	0
	8,13194	0	0	0

**BLOOD**	Group A	Group B	Group C	Group D
	MN	BDC	BDC+MPL16	BDC+CRL1506
	6,87	3,81	1,7	1,7
	6,78	3,39	2,81	1,7
	6,82	3,48	1,7	2,0

(MN group) malnourished mice without renourishment; (BDC group) renourished mice with a conventional balanced diet; (BDC+MPL16 and BDC+CRL1506 groups) renourished mice with a conventional balanced diet (BDC) and LAB.

Supplementary table 4: Data on the effect of LAB on intestinal cytokine levels after infection with *E. faecalis* 102.

**INTESTINAL TNF**	Group A	Group B	Group C	Group D
	MN	BDC	BDC+MPL16	BDC+CRL1506
	101,2	198,7	134,5	140,5
	111,2	197,5	135,8	142,4
	123,2	223,4	122,4	151,5
	121,4	225,6	123,8	149,7

**INTESTINAL IL-1β**	Group A	Group B	Group C	Group D
	MN	BDC	BDC+MPL16	BDC+CRL1506
	165,4	231,2	105,3	112,6
	166,3	234	103,2	115,2
	171,2	245,3	95,3	121,8
	173,8	244,1	93,1	122,9

**INTESTINAL IL-6**	Group A	Group B	Group C	Group D
	MN	BDC	BDC+MPL16	BDC+CRL1506
	99,8	156,7	212,3	189,3
	97,5	158,2	216,7	194,5
	104,6	163,1	231,2	221,4
	106,7	165,2	233,4	225,3

**INTESTINAL KC**	Group A	Group B	Group C	Group D
	MN	BDC	BDC+MPL16	BDC+CRL1506
	187,6	255,6	98,7	132,1
	188,3	253,4	99,3	133,7
	176,3	245,7	102,4	114,5
	178,4	247,6	103,9	117,8

**INTESTINAL IFN-γ**	Group A	Group B	Group C	Group D
	MN	BDC	BDC+MPL16	BDC+CRL1506
	114,5	234,5	376,4	354,6
	117,8	232,1	378,9	356

123,4	243,2	365,4	364,1
125,4	244,6	366,6	366,1

**INTESTINAL IL-17**	Group A	Group B	Group C	Group D
	MN	BDC	BDC+MPL16	BDC+CRL1506
	167,8	310,2	109,3	112,4
	166,5	311,4	111,3	115,6
	171,3	298,7	95,6	122,5
	172,5	299,1	97,1	123,7

**INTESTINAL IL-10**	Group A	Group B	Group C	Group D
	MN	BDC	BDC+MPL16	BDC+CRL1506
	234,5	398,1	498,7	489,7
	235,6	395,4	501,4	495,6
	243,8	388,2	513,2	476,5
	245,8	383,5	517,4	478,6

(MN group) malnourished mice without renourishment; (BDC group) renourished mice with a conventional balanced diet; (BDC+MPL16 and BDC+CRL1506 groups) renourished mice with a conventional balanced diet (BDC) and LAB.

Supplementary table 5: Data on the effect of LAB on serum cytokine levels after infection with *E. faecalis* 102

**SERUM TNF**	Group A	Group B	Group C	Group D
	MN	BDC	BDC+MPL16	BDC+CRL1506
	278,3	351,2	167,3	167,9
	275,4	354,3	165,4	168,3
	265,9	364,5	143,2	182,1
	266	366,4	144,8	183,4

**SERUM IL-β**	Group A	Group B	Group C	Group D

MN	BDC	BDC+MPL16	BDC+CRL1506
275,6	201,2	122,4	134,2
277,4	205,6	124,6	135,9
298,2	215,9	102,3	119,4
295,3	221,3	106,4	121,4

**SERUM KC**	Group A	Group B	Group C	Group D
	MN	BDC	BDC+MPL16	BDC+CRL1506
	283,4	250,1	113,4	145,6
	285,7	252,3	117,6	144,2
	299,3	245,4	122,4	132,4
	298,7	246,7	124,5	133,9

**SERUM IFN-γ**	Group A	Group B	Group C	Group D
	MN	BDC	BDC+MPL16	BDC+CRL1506
	122,3	254,3	341,2	312,5
	125,6	256,7	343,2	315,8
	135,6	261,4	331,8	298,7
	137,8	265,1	335,6	305,6

**SERUM IL-10**	Group A	Group B	Group C	Group D
	MN	BDC	BDC+MPL16	BDC+CRL1506
	432,1	451,2	178,3	213,4
	433,6	454,8	180,9	216,7
	412,7	467,1	193	198,5
	415,9	470,2	196,5	199,9

(MN group) malnourished mice without renourishment; (BDC group) renourished mice with a conventional balanced diet; (BDC+MPL16 and BDC+CRL1506 groups) renourished mice with a conventional balanced diet (BDC) and LAB.
